# A Case of Repeated Excision of the Inferior Dental and Gustatory Nerves for the Cure of Dental Neuralgia

**Published:** 1877-12

**Authors:** Theodore A. McGraw

**Affiliations:** Professor of Surgery, Detroit Medical College.


### ARTICLE VI.

### *A Case of Repeated Excision of the Inferior Dental and Gustatory Nerves for the Cure of Dental Neuralgia.*

[Read before the Detroit Medical and Library Association, by Theodore A. McGraw, M. D., Professor of Surgery, Detroit Medical College.]

The recurrence of neuralgia after its apparent cure by the excision of nervous trunks has been variously explained. In cases of centric origin, as in those cases where the pressure of an intercranial tumor excites pain in the extremities of a nerve, we can only wonder that operative procedures should give even the temporary relief which has sometimes followed excision. In cases of peripheral origin we have to assume, in explanation of the recurrent pain, either that the stump of the nerve has been affected by disease or compressed by cicatricial tissue, or that actual regeneration of the nervous trunk has been taking place. Modern researches have made it evident that this latter process is much more frequent than was once supposed. In the case which I will now relate I believe that the nerve was actually regenerated, connecting anew the central organ with some unknown spot of irritation, which excited the persistent pain.

In June, 1875, I was consulted by a patient from Gaines, Mich., on account of a neuralgia, of several years' duration, of the left lower jaw. He had been a man of very strong sexual passions, and he had indulged them without stint. He had been accustomed to use tobacco freely, was a hearty eater, but not, as I believe, addicted to drink. The teeth on the left side had all been taken out to relieve the intolerable pain. Nothing could be found in the mouth or face to account for the trouble.

On Feb. 20th, 1873, he had been operated on in Philadelphia by Prof. Gross, who trephined the horizontal portion of the bone and destroyed the nerve. The relief given by this operation continued over a year, when the pain again made its appearance in the jaw and for the first time in his tongue. It became in time so intense that he insisted upon having something done for its relief.

Assisted by his physician, Dr. Holly, of Vernon, and by some students from the college, on the 15th or June, 1875, I trephined the ramus of the jaw and cut out about half an inch of the inferior dental and gustatory nerves. His cheeks were very fat, and the bone was very dense and hard. The operation proved to be very difficult, and, as the trephine failed to make any impression on the ivory like bone, I was obliged to resort to the chisel and mallet. During this process the duct Steno became bruised, and a few days after the operation ulcerated though causing a salivary fistula. In other respects the patient did well and recovered rapidly, though it was many months before the fistula could be made to heal by appropriate treatment. He ceased, however, to suffer pain, and was well for a period of fourteen months. At the expiration of that time the neuralgia recurred. My attention had, in the mean time, been attracted to the following passage in Hyrtl's Typographische Anatomie:

"Richet relates that he had spoken with many persons who, on account of severe neuralgias of the teeth, had had this nerve (the auriculo-temporal) cut by a cobbler of the Rue des Feves, in Paris, who for thirty years had practiced this operation with immediate and permanent success, and to the annoyance of the medical faculty, with great renown. Richet convinced himself of the value of the treatment by operating upon a case of horribly painful and rebellious dental neuralgia, with the effect of the immediate relief of pain."

Acting on this suggestion, I laid the auriculo temporal nerve bare and divided it, but without any success in relieving the malady. A short time afterwards, at the request of the patient, whose sufferings were really atrocious, performed the operation recommended by Dr. Gross, and removed the whole alveolar process of the left side of the lower law. Not the slightest improvement followed the operation. Day and night the unfortunate man was tortured by the unbearable pain, which never altogether intermitted, and sometimes made him delirious with agony. Believing that the nerve had become regenerated I determined to divide it at a point still nearer its origin and to tear it loose from its connections. I hoped in this way to achieve the success which had followed the stretching of nerves in neuralgias of traumatic origin, by breaking up any inflammatory adhesions which might compress it or bind it to neighboring parts. I believe, too, that I might thus prevent its regeneration by changing its internal structure.

I operated on Sept. 28th, before the class of the Detroit Medical College, in the following manner. I made a T shaped incision with the horizontal portion just below the Zygoma—laid bare the bone and separated the upper and internal portion from the ramus with a chisel. This fragment, including the coronoid process, was turned up, leaving the nerves exposed as they came out from under the external pterygoid muscle. Pulling the nerves out with a blunt hook, I examined them carefully. They seemed to me to have become completely regenerated, passed down at the lower part of the wound where they had previously been divided without any break in their continuity until they were lost to sight. I grasped each separately with a pair of forceps and sought to pull them out from all their attachments. I had, however, miscalculated the strength of a living nervous cord, and found myself, after having made powerful traction in vain, obliged to cut them off with a pair of scissors, excising about three-quarters of an inch from each. The wound was then stuffed with carbolized lint and allowed to heal by granulation.

Healing occurred with great rapidity, and in a month the patient was ready to go home with but a small granulating spot remaining of the wound. There was, of course, paralysis of the eyelids, on account of the unavoidable division of the branches of the facial nerve. The neuralgia, however, was entirely relieved, and up to the present time he has had no signs of its recurrence. Whether the cure will be permanent, remains to be seen. I feared lest the operation at that point might cause anchylosis. This, however, is not the case. He has perfect motion and use of the jaw, and is entirely well of his salivary fistula. His long suffering has, however, broken his constitution, and his general health is wretched. He has, moreover, become addicted to the use of morphine, and has failed in many efforts which he has made to break the habit.

In view of the common recurrence of neuralgia after the excision of affected nerves and of the frequency of nervous regeneration as a cause of that recurrence, it would seem to me to be the duty of every operator to adopt such measures as would seem best adapted to prevent the growth of nervous tissue. Whether the stretching of nerves will accomplish the purpose remains to be seen; but it is at least worth a fair trial, and may be justified in practice, until experience proves its worthlessness or invention supplants it with better methods.—*Detroit Med. Journal.*

---

